# Assessment of the Frequency, Causes, Degree and Consequences of Violence against Health Workers in Psychiatric Institutions

**DOI:** 10.3390/healthcare12010084

**Published:** 2023-12-29

**Authors:** Zoran Jovanovic, Ana Opankovic, Srdjan Milovanovic, Jasmina Barisic, Tamara Nikolic Turnic, Dusan Djuric

**Affiliations:** 1 Department of Psychiatry, General Hospital Sabac, 15000 Šabac, Serbia; zoranjov73@gmail.com; 2 Clinical Centre of Serbia, Clinic for Psychiatry, Faculty of Medicine, University of Belgrade, 11000 Belgrade, Serbia; ana.opankovic@gmail.com (A.O.); or dr.srle011@gmail.com (S.M.); 3 Faculty of Medicine, University of Belgrade, 11000 Belgrade, Serbia; jasminabarisic@yahoo.com; 4 Department of Pharmacy, Faculty of Medical Sciences, University of Kragujevac, 34000 Kragujevac, Serbia; dusan.djuric@medf.kg.ac.rs; 5 N.A. Semashko Public Health and Healthcare Department, F.F. Erismann Institute of Public Health, I.M. Sechenov First Moscow State Medical University (Sechenov University), 119435 Moscow, Russia; 6 Institute for Rehabilitation, 11000 Belgrade, Serbia

**Keywords:** health care workers, violence, psychiatric institutions

## Abstract

(1) Background: The prevalence of workplace violence within the health sector varies between 50 and 88%. Depending on the health care environment, the percentages mentioned can be much higher. (2) The aim of this study was to determine the prevalence, characteristics, factors, and consequences of violence against healthcare workers (physicians, nurses, and technicians) in psychiatric institutions in the Republic of Serbia. Additionally, this study should validate the Serbian version of the aggression questionnaire, which could be a significant tool in recognizing and assessing any type of violence against health care workers in psychiatric institutions. (3) Methods: This study was designed as an observational questionnaire study that included 191 health workers (physicians, medical technicians, workers in kitchens or maintenance, and others) from three psychiatric institutions. As an instrument, this study validated and used the Serbian version of the aggression standardized questionnaire. We observed the primary and secondary outcomes of potential violence in psychiatric institutions against healthcare workers using different parameters. (4) Results: The internal consistency of each item as well as the instrument was very good (the mean Cronbach alfa = 0.91). A total of 104 of the participants never experienced physical violence, while more than five times that had 20 health workers (10.5%). We observed the statistical significance of gender, age, working status (permanent/limited) and professional status (physician/medical technician/worker etc.) on physical attack incidence. (5) Conclusions: The incidence of violence against healthcare workers is very high, especially in terms of physical assault and threats in the workplace. The majority of the victims were women who work as medical technicians, attacked by male patients with unknown motivation. A number of changes in the structure and organizational culture of the hospital are required. All hospital employees, employers, patients, and their families share responsibility for the creation of a safe workplace.

## 1. Introduction

Workplace violence refers to violent acts or threats of violence directed at employees in or out of the workplace, including verbal abuse, harassment, and physical assault and homicide. Violence in the workplace is becoming more common, and it is very difficult to assess the extent of this problem due to limited surveillance and an unsatisfactory level of awareness about it. Also, this problem can become a global phenomenon that disrupts peace and stability, which can ultimately put the health and well-being of the population at risk [1].

The prevalence of workplace violence within the health sector varies between 50 and 88% [2]. Depending on the health care environment, these percentages can be much higher. An example of an environment with a higher percentage of violence are emergency medicine clinics, where data from a meta-analysis of 14 studies shows that as many as 90% of employees were exposed to some type of violence [2]. During the pandemic, forms of violence against healthcare workers appeared, driven by the fear of SARS-CoV-2 transmission [3].

When it comes to psychiatric institutions, the prevalence of violence in a systematic literature review of 145 studies ranges between 8 and 76%, and these differences are due to the use of different research instruments, differences between the institutions where the examination was carried out, as well as the countries in which the studies were conducted. Various factors contribute to the occurrence of violence, so they are divided into three categories: factors related to patients, institutions, and employees. Within factors related to patients, we find those who are diagnosed with psychotic disorders, bipolar disorder, abuse of psychoactive substances, unmarried individuals, and younger ages. Employee-related factors include insufficiently trained or temporary staff, dissatisfaction with workplace management, the existence of burnout syndrome, and poor quality of interactions with patients. Finally, factors related to the institution itself are the overcrowding of the institution’s capacity, crowded places in psychiatric wards, walking time, unsafe environment, restrictive environment, lack of daily structure, smoking, and lack of privacy [4].

In a systematic review of 68 studies, the consequences of violence that health workers can experience are divided into seven categories: (1) physical, (2) psychological, (3) emotional, (4) changes in work ability, (5) changes in relationships with patients/quality of care, (6) social/general, and (7–8) financial. Psychological (depression, post-traumatic stress), emotional (anger, fear), as well as the impact on business functioning (sickness, job satisfaction) due to their frequent occurrence [5].

Violence affects people at all levels of society and can be present anywhere. In healthcare settings, violence could have many consequences for healthcare workers, patients, and the system at large. The ethics of violence can be pretty complex, and many risk factors are related to a lack of communication, systems, and environmental factors [5]. Violence against healthcare workers in any situation is inexcusable, especially when they are working around the clock to ensure that everyone receives the best treatment possible. The effect of violence harms healthcare employees’ physical and psychological well-being [6,7,8].

According to the previous meta-analysis, workplace violence mostly occurs in psychiatric departments and might lead to various negative impacts on health workers’ general and mental health, such as depression and anxiety [9]. In the USA, 25–85% of survey respondents reported an incident of physical aggression within the year prior to the survey, and statewide workers’ compensation findings indicated 2–7 claims due to assault per 100,000 employee hours [10]. In Germany, most of the violence still remains unreported and unexplored [11]. Nevertheless, 23% of interviewed hospital employees experienced at least one form of violence at the workplace [11]. In China, the incidence of workplace violence among psychiatric nurses is high, and the possible causes for Chinese psychiatric nurses suffering from violence include gender, education, working years, whether they are an only child, age, height, working hours, and the form of employment [12].

In the Republic of Serbia, there are 46 inpatient psychiatric institutions, 6 of which are special psychiatric hospitals, within which there are 41 beds per 100,000 inhabitants, with an additional 18 beds per 100,000 inhabitants within the psychiatric departments of general hospitals. The entire mental health sector has 6247 beds, of which 50% are located within large psychiatric hospitals. There are 12.6 psychiatrists, 2.3 psychologists, 1.6 social workers, and 21.6 nurses/technicians per 100,000 inhabitants [13,14,15,16,17]. In our healthcare institutions, there is no data on the prevalence of violence, factors associated with exposure to violence among healthcare workers, characteristics of experienced violence, or the consequences of such incidents on healthcare workers.

This study has several implications: workplace homicide is considered the third highest work-related cause of death in the United States and is the leading cause of death for women at work; it can harm physical and mental health; and it increases the costs of healthcare workers and facilities associated with losing a medical professional. In the Republic of Serbia, there is no actual study that examines the incidences and reasons for violence among healthcare workers in psychiatric institutions, which are the most specific regarding health care management. 

The aim of this study was to determine the prevalence, characteristics, factors, and consequences of violence against healthcare workers (physicians, nurses, and technicians) in psychiatric institutions in the Republic of Serbia. Also, this study should validate the Serbian version of the aggression questionnaire, which could be a significant tool in recognizing and assessing any type of violence among health care workers in psychiatric institutions.

## 2. Materials and Methods

### 2.1. Ethical Concerns

The research was conducted with the relevant directives in the field of clinical research ethics: Good Clinical Practice, the Helsinki Declaration, and the approval of the Ethics Committees of the Specialized Hospital Dr. Slavoljub Bakalovic, Vrsac, Serbia (number 01/901/2), the Specialized Psychiatric Hospital, Kovin, Serbia (number 04/561/9), and the Specialized Psychiatric Hospital Gornja Toponica, Nis, Serbia (number 08/3838/1). Voluntary written and informed consent was obtained from each participant before inclusion in the study.

### 2.2. Study Design and Protocol

This study was designed as an observational questionnaire and included 191 health workers (physicians, medical technicians, workers in kitchens or maintenance, and others) from three psychiatric institutions: the Specialized Hospital Dr. Slavoljub Bakalovic, Vrsac, Serbia; the Specialized Psychiatric Hospital, Kovin, Serbia; and the Specialized Psychiatric Hospital Gornja Toponica, Nis, Serbia. 

The main inclusion factors were a seniority of more than six months in the same institution, an age over 18 years, and voluntary consent to participate in the study. The study examines physical violence, verbal violence, and forms of verbal violence with a tendency to turn into physical violence. After they met all inclusion criteria, the participants obtained anamnestic and data by using the instrument of the Aggression Questionnaire (SerbAQ5) for a period of three months, from March to May 2023. 

### 2.3. Serbian Version of the Aggression Questionnaire (SerbAQ5)

The aggression questionnaire [18] is a standardized questionnaire for the use of which written confirmation was obtained from the author (Santiago Gascon, date 16 May 2023). The questionnaire was designed according to the principle of self-assessment by the respondent, which analyzes different forms of aggression. Participants were asked to indicate experiences with aggressive behavior in the workplace in the previous 12 months. The questions and answers in the questionnaire are based on the principle of the Likert scale: 0: never; 1: never, but I am a witness to what is happening with others; 2: once; 3: two or more times; 4: more than five times. In the case of positive answers in terms of the existence of aggression, the respondents were asked to describe the characteristics of violence and aggressors, regardless of how much previous professional knowledge the respondent had about the concept of aggression. 

The first part (historical data) of the questionnaire presents basic socio-demographic data as well as data on length of employment in healthcare, length of work in the current position, work status, and absence from work in the previous 12 months.

The second part (aggression data) of the questionnaire consists of three sub-parts related to workplace aggression. The first sub-part is aimed at understanding the event in which the violence occurred. The questions are aimed at detecting exposure to certain types of violent attacks, threats, and insults, and this part is organized so that the respondents answer on a Likert scale whether they have ever experienced violence during their working career. Respondents were also asked whether they were specially trained in order to protect themselves from situations in which violence may occur, as well as whether they have the support of health institutions in cases of violence directed at them. The second sub-part of the unit on aggression was filled in if the respondent had experienced any kind of violence. In this part, the questions are focused on the consequences that followed the incident, its duration, and the appeal for help.

The third sub-part was filled in by respondents who experienced physical violence, with a focus on the identification, characteristics, and motive of the attacker, on the type of physical aggression, the part of the body that was attacked, the result of the aggression, whether medical help was provided, whether they were absent from work due to attacks, as well as ultimately whether there was an appeal to a legal entity for the attack. The questionnaire is anonymous, in no way reveals the potential identity of the victim or aggressor, and is used exclusively for scientific research purposes [18].

### 2.4. Validation of the Serbian Version of the Aggression Questionnaire (SerbAQ5)

The SerbAQ5 was translated from English to Serbian following the WHO’s guide for translation and adaptation of instruments to be sure of linguistic and cultural equivalence between the English and Serbian versions. The translation process was performed by forward and backward translation, a review of cognitive interviews, and additional cognitive interviews. The final step was proofreading and finalizing the Serbian version of SerbAQ5 [18].

For the validation of the Serbian version of SerbAQ5, participants were randomly recruited from a study sample during a clinical study. All participants met the inclusion criteria defined prior to this article. Test–retest reliability between the first and second responses to the questionnaire was calculated for all items based on the intraclass correlation coefficient (ICC). An ICC below 0.50 reflected poor reliability, between 0.50 and 0.75 reflected moderate reliability, and above 0.75 reflected good reliability. 

After that, we estimated the internal consistency of the instrument, where we measured the reliability of the items and separate domains. The Cronbach alfa coefficient, with a range from 0 to 1, was calculated. Cronbach’s α values ranged between excellent (≥0.9), good (0.8–0.9), acceptable (0.7–0.8), questionable (0.6–0.7), poor (0.5–0.6), and unacceptable (≤0.5).

### 2.5. Test–Retest Reliability and Validation of the SerbAQ Instrument

The internal consistency of each item as well as the Cronbach alfa for the instrument is very good (the mean Cronbach alfa is 0.91). Also, the IIC for each is high and above 0.75, which indicates that all items meet the criteria and standards for internal consistency and adequate tooling. 

### 2.6. Analysis of Primary and Secondary Outcomes using a Validated Instrument

In its analysis, the study evaluates the primary and secondary outcomes of potential violence in psychiatric institutions against healthcare workers. The primary outcomes are the frequency and type of violence, duration of violence, potential cause of violence, place and type of institution in relation to the type of violence, absence from work in the previous 12 months, as well as all other general socio-epidemiological characteristics of the aggressor and the person who is experiencing some type of aggression.

In the second part of the analysis, the study will deal with the examination or identification of the characteristics and motives of the attackers. In addition, a special aspect is the type of physical aggression, the part of the body that was attacked, the result of the aggression, and whether medical assistance was provided. Among the most important secondary outcomes are the consequences of aggression, the analysis of the consequences that followed the incident, its duration, and the appeal for help. 

### 2.7. Statistical Analysis and Sample Size Calculation

The calculation of the total sample is based on the results of a previously published study of a similar design [18]. The Chi squared test is used for the calculation twofold, assuming an alpha error of 0.05 and study power of 0.8 (beta error of 0.2) and using the appropriate computer program for calculating the power of the study by type of cross-sectional study and prediction calculation. 

All data are presented as absolute numbers or as frequency in percent (%). The assessment of the normality of the data distribution is based on the Shapiro–Wilk test. In the methods of analytical statistics for the comparison of numerical characteristics of observations between groups, the Chi Square test is used to evaluate the distribution of certain categorical variables in relation to other variables. 

The chosen level of statistical significance, that is, the probability of a type I error, is 0.05. All obtained results are presented tabularly. A value of *p* < 0.05 is considered statistically significant, and a value of r < 0.01 is highly significant. The validity and reliability of the used version of the questionnaire are calculated using a standard validation process and confirmatory factor analysis. All analysis is conducted in a licensed statistical program, SPSS version 26 (IBM SPSS for Macintosh).

## 3. Results

### 3.1. Basic Demographic and Anamnestic Characteristics of the Study Population

Among the study population, which included 191 participants, women (66.0%) with the age categories of 36–54 years and married were predominant. Most of them (46.1%) had two children and worked for more than 10 years in the health system (Table 1). Most of them worked in permanent positions as medical technicians, with very rare absences from work (Table 1).

Based on the presence of physical violence, 87 participants experienced attacks in their history, while 104 participants had no experience of physical aggression at work (Table 1). The differences between these two groups regarding the basic demographic data are presented in Table 1. 

### 3.2. Prevalence of Different Types of Violence among the Study Population

From the total number of participants (n = 191), 104 of them never experienced physical violence, while more than five times that had even 20 health workers (10.5%) (Table 2). Verbal violence was more frequent, and once experienced 23, and more than five times 53 health workers. Additionally, the treats were similarly frequent as objections against health workers, and only 42.9% of participants never had that experience. The complaints had a lower frequency among the study population, and 150 (78.5%) had not experienced this type of violence (Table 2). Definitely, the prevalence of any type of violence in our study population of health workers is high, and above half of the participants were affected by some type of aggression against them (Table 2).

### 3.3. Characteristics of Aggressors and Violence among Health Workers

In this part of the study, we evaluated the characteristics of aggression and attackers according to the answers of health workers and their experiences. All data regarding that are presented in Table 3. In the group of participants who had a physical attack in their history, most of them reached out for help after the incident (98.8%). Also, they had symptoms that lasted for 1–7 days, but there were also participants who had symptoms for more than 90 days (2.3%) (Table 3). Regarding the type of symptoms, they were very different, from anxiety to insomnia, irritability, and loss of memory, which also lasted for many days (Table 3). The aggressor was a patient in most of the cases (87.4%) and not conspicuous (79.2%). The main reason for any incident against health workers was unknown (71.3%), and the other reasons were related to the health care system, rules, admission and discharge, or prescribed/applied therapy (Table 3). Physical attack consisted of scratching/pushing in most of the cases (26.4%) in the region of the head and neck (26.3%). The consequences of the attack were based on scars (17.2%), bruises (2.4%), and medical advice and aid (28.7%) (Table 3). 

### 3.4. Association between Basic Characteristics of Health Workers and Characteristics of Violence under Them

In this study, we aim to find a potential relationship between the basic characteristics of health workers and the characteristics of violence against them. The results of this analysis are presented in Table 4. We observe the statistical significance of gender, age, working status (permanent/limited) and professional status (physician/medical technician/worker etc.) on physical attack incidence (Table 4). Moreover, position experience is statistically significant in determining the reason for the attack; marital status, having children, working experience, and professional status are connected with the request for medical help after the incident. Working experience was highly statistically significant when connected with medical help and having children in their own family. Also, the reason for the attack was highly connected with the position and working experience of health workers. Interestingly, none of the tested cases were significant in the type of attack or requesting the help of a legal entity (Table 4). All the variables mentioned in Table 4 clearly describe the most important risk factors for violence among healthcare workers in our three psychiatric institutions, and most of them have a statistically confirmed connection with characteristics of physical attack. 

## 4. Discussion

Health workers are at high risk of violence all over the world, and approximately 30% of them suffer from some type of violence at some point in their careers. There is a different category of health workers: those who are directly involved in patient care, emergency units, additional medical activities, and rare interactions with patients. In all of those situations, violence against any health care worker is unacceptable. There are many strategies regulated by the World Health Organization and each institution, but all these procedures are still not sufficient to reduce the total amount of any type of violence [19,20,21]. Unsurprisingly, psychiatric workers have the highest incidence rate of non-fatal injuries, at more than 10 times the rate of other hospitals [22,23]. Long-term nursing and residential care facilities are experiencing increased rates of violence, about 75% higher than the rate of violence in hospitals [24,25]. 

In our study, from the total number of participants (n = 191), 104 of them never experienced physical violence, while more than five times there were even 20 health workers (10.5%) (Table 2). Verbal violence is more frequent, once experienced in 23 and more than five times in 53 health workers. Also, the treats were similarly frequent as an objection against health workers, and only 42.9% of participants never had that experience. The complaints have a lower frequency among the study population, and 150 (78.5%) had not experienced this type of violence (Table 2). Definitely, the prevalence of any type of violence in our study population of health workers is high, and above half of the participants were affected by some type of aggression against them (Table 2).

Previous studies reported similar results. Alsaleem A conducted a study in which a total of 738 healthcare workers responded (92% response rate). The mean age was 31 ± 7.7 years (range 21–60), and the majority (64.9%) were females and 69.4% were Saudis. More than half (57.5%) had experienced some workplace violence at least once. Verbal assaults and slaps were the most common form of workplace-related violence (58%) [26].

In a study conducted in 2009 in Al-Hassa, Saudi Arabia, it was found that about 28% of the 1091 workers studied had been exposed to at least one violent event in the previous year; 92.1% of this was emotional and 7.9% was physical [27].

Another study conducted in the emergency departments of Palestinian hospitals in 2013 showed that around 35.6% of staff had been exposed to physical assaults and 71.2% were exposed to nonphysical assaults [28].

In the group of participants who had a physical attack in their history, most of them reached out for help after the incident (98.8%). Also, they had symptoms that lasted for 1–7 days in most of them, but there was also a participant who had symptoms for more than 90 days (2.3%) (Table 3). Regarding the types of symptoms, they are very different, from anxiety to insomnia, irritability, and loss of memory, which also lasted for many days (Table 3). The aggressor was a patient in most of the cases (87.4%) and not conspicuous (79.2%). The main reason for any incident against health workers was unknown (71.3%), and the other reasons were related to the health care system, rules, admission and discharge, or prescribed/applied therapy (Table 3). Physical attack consisted of scratching/pushing in most of the cases (26.4%) in the region of the head and neck (26.3%). The consequences of the attack were based on scars (17.2%), bruises (2.4%), and medical advice and aid (28.7%) (Table 3). Literature data also suggested that the prevalence of workplace violence in general was 58.7%; physical violence was 20.8%; verbal violence was 66.8%; and sexual harassment was 10.5% [10].

Definitely, aggression could be direct behaviors such as hitting, kicking, biting, and pushing, to name a few. Additionally, aggression can take on an indirect form like teasing, bullying, spreading rumors, name-calling, or ignoring someone. One of the previous studies conducted by Falcao de Oliviera described the characteristics of aggressors [24]. Regarding the aggressors, the largest percentages were male aggressors with only primary school (45.9%), illegal drug users (60.4%), and self-employed/freelance workers (72.4%). Regarding the classification of the injuries (according to the Brazilian Penal Code), there were 971 cases of injuries considered slight, 23 of severe injuries, and 6 of very severe injuries [24].

In our study, since most of the victims were women and most of the aggressors were male patients, there are some specificities about the violence against women. Gender stereotypes are sometimes used to attempt to justify violence against women. Cultural norms often dictate that men are aggressive, controlling, and dominant, while women are docile, subservient, and rely on men as providers. The term violence against women means any act of gender-based violence that results in, or is likely to result in, physical, sexual, or psychological harm or suffering to women, including threats of such acts, coercion, or arbitrary deprivation of liberty, whether occurring in public or private life [25].

In the end, we evaluated the reasons and inductors of violence among the study population. We observed the statistical significance of gender, age, working status (permanent/limited) and professional status (physician/medical technician/worker etc.) on physical attack incidence (Table 4). Also, position experience was statistically significant in determining the reason for the attack; marital status, having children, working experience, and professional status were connected with requesting medical help after the incident. Interestingly, none of the tested carriers were significant in the type of attack or requesting the legal entity (Table 4).

Chinese scientists examined the factors that influence workplace violence, and some of them were untrained workers, low communication skills, and the absence of response tactics [12]. Another meta-analysis, which included 27 relevant publications, confirmed that lack of information, insufficient personnel and equipment, and communication breakdowns increase the risk of violent behavior in healthcare services [11].

The circumstances under which care is provided in healthcare settings make health care professionals especially vulnerable to occupational violence. The factors that account for the increased incidence of violence towards healthcare professionals can be summarized as follows: Healthcare professionals come into direct contact with a wide range of people who are under stress due to pain or illness [19], nursing staff care for people who are confused or emotionally unstable [20], sometimes healthcare staff has to provide care in secluded areas and during diagnostic or therapeutic interventions [21], and nursing staff often performs interventions that require close physical contact [22], healthcare staff comes into contact with patients’ families who are often under intense emotional charge due to severe trauma, mental disorder, bereavement, etc. [22,29].

The limitation of this study lies in the cross-sectional design of the study, which sometimes could not be sufficient to recognize and follow the victims and aggressors. On the other hand, we use strictly selected specialized psychiatric institutions where there is a specific working place for all health care workers and where it is very hard to obtain any information about the potential violence. Because of these facts, we consider that to be the main strength of our study.

This paper provides new information about the prevalence, characteristics, and potential factors of violence in psychiatric institutions and among healthcare workers, since this population is under specific working conditions. Specifically, with regard to workplace violence within the health sector, if appropriate protective measures are not taken, this problem can become a global phenomenon that disrupts peace and stability in such environments, which can ultimately put the health and well-being of the population at risk.

This study highlights the importance of preventing violence against healthcare workers in psychiatric institutions in Serbia and points to the necessity of interventions aimed at protecting employees in the form of training, providing physical protection measures, early identification of employees exposed to verbal violence as a potential precursor to physical violence, the formation of an employee protection policy, and greater support from superiors.

## 5. Conclusions

The incidence of violence against healthcare workers is very high, especially in terms of physical assault and threats in the workplace. Most of the victims are women who work as medical technicians and are attacked by male patients of unknown motivation. A number of changes in the structure and organizational culture of the hospital are required. All hospital employees, employers, patients, and their families share responsibility for the creation of a safe workplace. Future directions in research on this topic must be focused on finding strategies for violence among healthcare workers and improving the healthcare system and facilities in psychiatric institutions. Violence against health care workers in psychiatry is on the rise; they deserve protection and better security at the workplace.

## Figures and Tables

**Table 1:** Basic socio-demographic and anamnestic data of the study population (n = 191). Statistical analysis is performed using the Chi square test. The high significance is set equal to or below 0.01 (**).

Variables	All Participants (n = 191)		Group with Physical Violence (n = 87)		Group without Physical Violence (n = 104)		*p*
Gender	F 126 (66.0%)	M 65 (34.0%)	F 48 (55.2%)	M 38 (43.7%)	F 77 (74.0%)	M 27 (26.0%)	0.001 **
Age (category in years)	<25 12 (6.3%) 26–35 35 (18.3%) 36–54 97 (50.8%) >55 47 (24.6%)		<25 10 (11.5%) 26–35 37 (42.5%) 36–54 19 (21.8%) >55 21 (24.2%)		<25 20 (9.7%) 26–35 42 (40.3%) 36–54 26 (25.0%) >55 26 (25.0%)		0.003 **
Marital status	Single 37 (19.4%) Married 117 (61.3%) In relationship 37 (19.4%)		Single 16 (18.4%) Married 47 (54.0%) In relationship 23 (26.4%)		Single 20 (19.2%) Married 70 (67.3%) In relationship 14 (13.5%)		0.001 **
Number of children	None 49 (25.7%) One 37 (19.4%) Two 88 (46.1%) Three 16 (8.4%) Four 1 (0.5%)		None 23 (26.4%) One 19 (21.8%) Two 38 (43.7%) Three 5 (5.7%) Four 1 (1.1%)		None 26 (25.0%) One 17 (16.3%) Two 50 (48.1%) Three 11 (10.6%)		0.345
Duration of working in the HS	1–2 years 13 (6.9%) 11–15 years 28 (14.8%) 16–20 years 19 (10.1%) 3–5 years 23 (12.2%) 6–10 years 25 (13.2%) 7–11 months 6 (3.2%) >20 years 75 (39.7%)		1–2 years 6 (6.9%) 11–15 years 12 (13.8%) 16–20 years 12 (13.8%) 3–5 years 7 (9.0%) 6–10 years 8 (9.2%) 7–11 months 1 (1.1%) >20 years 40 (46.0%)		1–2 years 7 (6.9%) 11–15 years 16 (15.7%) 16–20 years 7 (6.9%) 3–5 years 16 (15.7%) 6–10 years 17 (16.7%) 7–11 months 5 (4.9%) >20 years 34 (33.3%)		0.004 **
Duration of working in the current position	1–2 years 28 (14.9%) 11–15 years 21 (12.0%) 16–20 years 17 (9.1%) 3–5 years 39 (20.6%) 6–10 years 27 (14.3%) 7–11 months 7 (3.8%) >20 years 45 (23.8%)		1–2 years 16 (18.6%) 11–15 years 10 (11.6%) 16–20 years 8 (9.4%) 3–5 years 13 (15.1%) 6–10 years 11 (12.7%) 7–11 months 2 (2.5%) >20 years 24 (27.7%)		1–2 years 12 (11.6%) 11–15 years 11 (10.7%) 16–20 years 8 (7.9%) 3–5 years 26 (25.2%) 6–10 years 16 (15.5%) 7–11 months 5 (4.9%) >20 years 21 (20.4%)		0.231
Working status	Permanent 173 (90.6%) Limited 18 (9.4%)		Permanent 84 (96.6%) Limited 2 (2.3%)		Permanent 88 (84.6%) Limited 16 (15.4%)		0.433
Professional status	Physician 8 (4.2%) Physician with specialization 27 (14.1%) Medical technician 123 (64.4%) Other 8 (4.2%) Maintenance worker 5 (2.6%) Kitchen worker 3 (1.6%)		Physician 4 (4.6%) Physician with specialization 15 (17.2%) Medical technician 62 (71.3%) Kitchen worker 3 (3.4%)		Physician 4 (3.8%) Physician with specialization 12 (11.5%) Medical technician 61 (58.7%) Other 8 (7.7%) Maintenance worker 5 (4.8%)		0.543
Absence in the last 12 months (yes/no)	Yes 51 (26.7%)	No 140 (73.3%)	Yes 21 (24.1%)	No 65 (74.7%)	Yes 30 (28.8%)	No 74 (71.2%)	0.134
Reason for absence	Unknown 16 (8.4%) Disease 31 (16.2) Postpartum 5 (2.6%)		Unknown 2 (2.3%) Disease 17 (19.5%) Postpartum 2 (2.3%)		Unknown 14 (13.5%) Disease 14 (13.5%) Postpartum 2 (2.6%)		0.346
Absence in the last 12 months (days)	1–14 days 22 (11.5%) 15–30 days 13 (6.8%) 30–90 days 9 (4.7%) >90 days 9 (4.7%)		1–14 days 10 (11.5%) 15–30 days 6 (6.9%) 30–90 days 5 (5.7%) >90 days 0 (0%)		1–14 days 12 (11.5%) 15–30 days 7 (6.7%) 30–90 days 4 (3.8%) >90 days 7 (4.6%)		0.331

**Table 2:** Prevalence of different types of violence against health workers experienced in previous times among the study population (n = 191). Statistical analysis is performed using the Chi square test. The statistical threshold is set at 0.05.

Have You Ever Experienced:					
	Never Number (%)	Never, but I Attended Number (%)	Once Number (%)	More Than Twice Times Number (%)	More Than Five Times Number (%)
Physical violence	104 (54.0)	23 (12.0)	24 (12.6)	20 (10.5)	20 (10.5)
Verbal violence	61 (31.9)	20 (10.5)	23 (12.0)	34 (17.8)	53 (27.7)
A threat	82 (42.9)	17 (8.9)	20 (10.5)	33 (17.3)	39 (20.4)
An objection	88 (46.1)	22 (11.5)	40 (20.9)	23 (12.0)	18 (9.4)
The complaints	150 (78.5)	13 (6.8)	20 (10.5)	6 (3.1)	2 (1.0%)
Trained in terms of defense against violence	Yes 117 (61.0%) 74 No (39.0%)				
Institution’s support in terms of violence	Yes 118 (62.0%) 73 No (38.0%)				

**Table 3:** Characteristics of violence, aggressor, and consequences of violence in the group that experienced physical attack (n = 87). Statistical analysis is performed using the Chi square test. The statistical threshold is set at 0.05 (*); high significance is set equal to or below 0.01 (**).

Variables	Group Experienced Physical Attack (n = 87)		Chi Squared Test *p*-Value
Did you reach out to anyone for help after the incident, and if so, why?	Yes 86 (98.8%)	No 1 (1.2%)	0.001 **
These symptoms persisted for (days)	1–7 days 14 (16.1%) 8–14 days 3 (3.4%) 15–30 days 5 (5.7%) 31–90 days 4 (4.6%) >90 days 2 (2.3%)		0.001 **
Did you have any feelings or experiences after the incident?	Yes 47 (54.4%)	No 40 (46.6%)	0.565
Did you have any of the following feelings or experiences after the incident?	Anxiety 8 (9.2%) Insomnia 3 (3.4) Fair 9 (10.5%) Mistrust 1 (1.1%) Sadness 7 (8.0%) Difficulty in concentration 1 (1.1%) Irritability 2 (2.3%) Disturbing memories 5 (5.7%)		0.477
These symptoms lasted (days)	1–7 days 14 (16.1%) 8–14 days 6 (6.9%) 15–30 days 9 (10.3%) 31–90 days 3 (3.4%) >90 days 3 (3.4%)		0.761
Who made the attack	Patient 80 (87.4%) Companion of the patient 1 (1.1%)		0.001 **
Attacker Characteristics	Conspicuous 19 (21.8%) Not conspicuous 68 (79.2%)		0.001 **
The motive for the attack	Dissatisfaction with the service 5 (5.7%) Admission and discharge of the patient 4 (4.6%) House Rules 7 (8.0%) Alcohol consumption 4 (4.6%) Prescribed/applied therapy 5 (5.7%) Unknown motive 62 (71.3%)		0.134
Physical aggression consisted of the following	Scratching/pushing 23 (26.4%) throwing objects 5 (5.7%) suffocation attempt 3 (3.3%) hitting 14 (16.1%)		0.023 *
Part of the body was attacked	Head 25 (26.3%) upper extremities 14 (15.9%) lower extremities 1 (1.1%) Trunk 7 (8%) Neck 2 (2.3%)		0.045 *
The result of the physical aggression	without lesion 29 (33.0%) bruises 2 (2.4%) scars 15 (17.2%) contusions 1 (1.1%) cuts 1 (1.1%) other 3 (3.4%)		0.011 *
Requesting medical help	First aid and treatment 1 (1.1%) First aid 13 (14.9%) Advice 25 (28.7%)		0.044 *
Have you been absent from work because of the above-mentioned injuries?	Yes 2 (2.3%)	No 49 (56.3%)	0.001 **
Requesting the legal entity (lawyer, court)	Yes 2 (2.3%)	No 49 (56.3%)	0.033 *

**Table 4:** Results of the Chi squared test for determining the significant parameter in type and reason for physical violence [n = 191]. The statistical threshold is set at 0.05 (*); high significance is set equal to or below 0.01 (**).

Chi Square Test	Physical Attack			Reason			Type			Medical Help			Law Help		
	Value	df	*p*	Value	df	*p*	Value	df	*p*	Value	df	*p*	value	df	*p*
Gender	11.51	4	0.02 *	14.61	10	0.062	12.31	9	0.123	2.04	3	0.519	0.47	2	0.795
Age	25.72	12	0.012 *	18.34	30	0.821	31.78	27	0.605	3.04	9	0.892	2.60	6	0.807
Marital status	10.82	8	0.229	18.35	20	0.355	16.71	18	0.542	13.05	6	0.042 *	8.72	4	0.065
Children	17.38	16	0.361	38.88	40	0.520	31.42	36	0.686	26.33	12	0.010 **	11.19	8	0.191
Working experience	27.71	28	0.480	75.65	70	0.301	61.67	63	0.524	39.17	21	0.009 **	18.80	14	0.173
Position experience	33.68	28	0.211	100.27	70	0.010 **	80.40	63	0.069	25.36	21	0.232	17.84	14	0.214
Working status	9.68	4	0.046 *	6.38	10	0.782	12.26	9	0.199	0.352	3	0.950	0.354	2	0.838
Professional status	31.25	20	0.021 *	41.38	50	0.802	46.36	45	0.416	26.12	15	0.037 *	12.53	10	0.251

## Data Availability

The data are available from the corresponding authors upon reasonable request.
